# Fimasartan for Remodeling after Myocardial Infarction

**DOI:** 10.3390/jcm8030366

**Published:** 2019-03-15

**Authors:** Byung-Kwan Lim, Jin Joo Park, Sung-Ji Park, You-Jung Lee, Jin-Sook Kwon, Eun-Ji Kim, Dong-Ju Choi

**Affiliations:** 1 Department of Biomedical Science, Jungwon University, Goesan-gun 28024, Korea; bkwan.lim@gmail.com; 2 Department of Internal Medicine, College of Medicine, Seoul National University Bundang Hospital, Seongnam 13620, Korea; jinjooparkmd@gmail.com (J.J.P.); until10@hanmail.net (E.-J.K.); 3 Division of Cardiology, Department of Internal Medicine, Cardiovascular Imaging Center, Heart Vascular Stroke Institute, Samsung Medical Center, Sungkyunkwan University School of Medicine, Seoul 06351, Korea; tyche.park@gmail.com (S.-J.P.); eelulee@daum.net (Y.-J.L.); 4 Division of Cardiovascular and Rare Disease, Korea National Institute of Health, Osong, Cheongju, Chungbuk 28159, Korea; gonkjs@hanmail.net

**Keywords:** fimasartan, angiotensin receptor blocker, myocardial infarction, cardiac remodeling, microarray

## Abstract

An angiotensin receptor blocker (ARB) mitigates cardiac remodeling after myocardial infarction (MI). Here, we investigated the effect of fimasartan, a new ARB, on cardiac remodeling after MI. Sprague–Dawley rats were assigned into 3 groups: surgery only (sham group, *n* = 7), MI without (MI-only group, *n* = 13), and MI with fimasartan treatment (MI + Fima group, *n* = 16). MI was induced by the permanent ligation of the left anterior descending artery. Treatment with fimasartan (10 mg/kg) was initiated 24 h after MI and continued for 7 weeks. Rats in the MI + Fima group had a higher mean ejection fraction (66.3 ± 12.5% vs. 51.3 ± 14.8%, *P* = 0.002) and lower left ventricular end-diastolic diameter (9.14 ± 1.11 mm vs. 9.91 ± 1.43 mm, *P* = 0.045) than those in the MI-only group at 7 weeks after MI. The infarct size was lower in the MI + Fima than in the MI group (*P* < 0.05). A microarray analysis revealed that the expression of genes related to the lipid metabolism and mitochondrial membrane ion transporters were upregulated, and those involved in fibrosis and inflammation were downregulated by fimasartan. Fimasartan attenuates cardiac remodeling and dysfunction in rats after MI and may prevent the progression to heart failure after MI.

## 1. Introduction

Heart failure (HF) is the leading cause of hospitalization worldwide, and its incidence has been steadily increasing [1]. Ischemic cardiomyopathy is the most common etiology of HF [2], and the cardiac remodeling after myocardial infarction (MI) is the underlying pathophysiologic mechanism.

In patients after MI, the renin-angiotensin-aldosterone system (RAAS) activity is substantially increased [3]. Angiotensin II induces myocardial hypertrophy, fibrosis, and apoptosis after binding to the angiotensin II type I receptor (AT1R), and AT1R antagonists inhibit the fibrosis in the myocardium after MI [4]. Thus, the use of angiotensin receptor blockers (ARBs) exerts a beneficial effect on the cardiac remodeling and mitigates the progression to HF [4,5].

Fimasartan (BR-A-657; BR-A-657-K; Kanarb^®^) is a new ARB with a high selectivity for the AT1R subtype. Fimasartan inhibited the contraction of isolated rabbit thoracic aorta compared with other ARBs [6]. It also showed protective effects in an acute MI model [7]; fimasartan significantly reduced the infarct size after ischemia and reperfusion (I/R) injury and prevented mitochondrial dysfunction by inhibiting Ca^2+^ overload during acute reoxygenation [8,9].

Nonetheless, it is unknown whether fimasartan has any protective effect on cardiac remodeling after MI. In this study, we sought to evaluate the effect of fimasartan on cardiac remodeling after MI in Sprague–Dawley rats.

## 2. Materials and Methods

### 2.1. Animal Experiment

**Figure 1:**
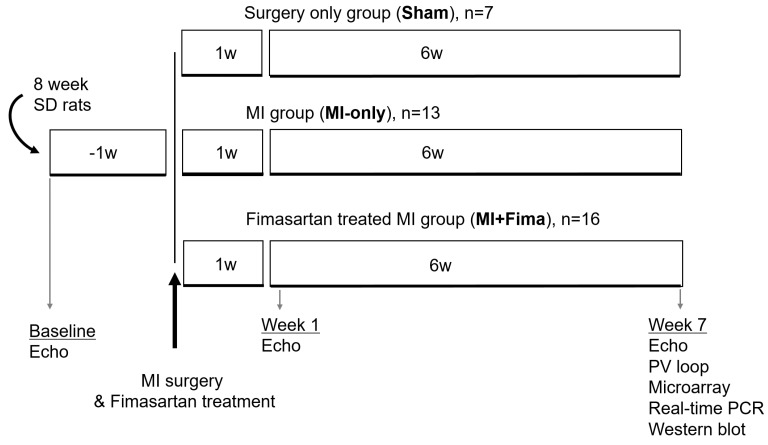
A schematic diagram of the experimental protocol. echo, echocardiography; Fima, fimasartan; MI, myocardial infarction; PCR, polymerase chain reaction; SD, Sprague–Dawley; PV, pressure–volume; w, week.

Eight-week-old male Sprague–Dawley rats weighing 200–250 g each were used. The study protocols conformed to the Guide for the Care and Use of Laboratory Animals published by the National Institutes of Health (Publication 85–23, revised 1996). All procedures were reviewed and approved by the Institutional Animal Care and Use Committee of Seoul National University Bundang Hospital (BA1206-107/049-01). The dimmers were used in rat rooms to create twilight periods between the light and dark cycles. A room temperature between 20–26 °C was maintained. Rats with normal left ventricular (LV) function were assigned to the surgery-only group (sham group, *n* = 7), the MI without fimasartan treatment group (MI-only group, *n* = 13), and the MI with fimasartan treatment group (MI + Fima group, *n* = 16) (Figure 1).

For the introduction of MI, the rats were intubated with a 16-gauge catheter after an induction with 5% isoflurane followed by maintenance anesthesia with 1.5% isoflurane. After vertical thoracotomy and pericardiectomy, the heart was exposed and MI was induced by the snaring of the left anterior descending artery with a 6–0 silk suture. The surgical site was disinfected using “povidone-iodine”, and ibuprofen was orally delivered to relieve pain after surgery. Fimasartan (Kanarb, Boryung Inc., South Korea) was dissolved by 0.5% carboxymethyl cellulose and administered orally (10 mg/kg/day) for 7 weeks. The sham and MI-only groups were treated with 0.5% carboxymethyl cellulose only. Echocardiography was performed at baseline and at 1 and 7 weeks of treatment. After the final echocardiography, hemodynamic measurements were performed, followed by organ harvesting. For the histologic examinations, the tissue samples were stained with hematoxylin and eosin and Masson trichrome staining.

### 2.2. Echocardiography

Echocardiography was performed under anesthesia with a ketamine (100 mg/kg) and xylazine (5 mg/kg) mixture using a commercially available ultrasound system (Acuson Sequoi 512C system, Siemens, Mountain View, Santa Clara County, CA, USA) with a linear array transducer (14 MHz). The chest was shaved, and the animal was positioned on a heating pad in a supine position. A single-channel electrocardiogram was obtained on the imaging system. Two-dimensional echocardiographic loops and M-mode images of three consecutive beats were obtained. The imaging depth was adjusted to 30 or 40 mm, resulting in a temporal resolution of 100–150 Hz. All measurements and calculations were performed according to American Society of Echocardiography standards [10]. For the M-mode recordings, the parasternal long-axis view was used and the following dimensions were measured: left ventricular end-diastolic diameter (LVEDD), left ventricular end-systolic diameter (LVESD), interventricular septum thickness (IVSd), and left ventricular posterior wall thickness (LVPWd). The ejection fraction (EF) was calculated [10]. All acquisitions were performed by the same operator, while the off-line analysis was conducted by an echocardiography specialist who was blinded to the allocated groups.

### 2.3. Hemodynamic Measurements

After the echocardiography, the rats were intubated with a 16-gauge catheter after induction with 5% isoflurane. Anesthesia was maintained with 1.5% isoflurane, and the rats were placed in the recumbent position on a heating pad with a rectal probe connected to a thermos regulator. The animals were ventilated with a constant-pressure ventilator (Harvard Co., Boston, MA, USA) at 75 breaths/min using room air. An anterior thoracotomy was performed, and a small apical stab was made to expose the LV apex. After the stabilization of the LV apex with a 27-gauge needle, a microtip pressure–volume (P–V) catheter (SPR-838, Millar Instruments; Houston, TX, USA) was inserted retrogradely into the LV cavity along the cardiac longitudinal axis until stable P–V loops were obtained [3,11]. The abdominal wall was opened, and the inferior vena cava (IVC) and portal vein were exposed. A snare suture was placed to modulate the rapid IVC obstruction. All loops were acquired after 20 min of stabilization with the ventilator turned off for 5–10 s. The sampling rate was 1,000/s using the ARIA P–V conductance system (Millar Instruments) coupled to a PowerLab 16/30A/D converter (AD Instruments, Mountain View, CA, USA) and a personal computer. All data were analyzed as previously reported [3]. Ten to 20 successive cardiac cycles were obtained over 5 s, from which the end-systolic pressure volume relation slope, maximum first derivative of ventricular pressure with respect to time (dP/dt_max_)–end diastolic volume relation, and end-diastolic pressure volume relation slope were derived.

### 2.4. Western Blot Analysis

For the western blot analysis, a non-infarcted zone of 50 mg of ventricle heart was lysed in a RIPA (Radioimmunoprecipitation assay) buffer (50 mM Tris (pH 8.0), 0.1% SDS, 1% NP40, 150 mM NaCl, and 0.5% sodium-deoxycholate). Ten-microgram aliquots of the total heart extract were loaded onto 10% sodium dodecyl sulfate–polyacrylamide gel electrophoresis gels, processed for 4 h at 100 Volt, and transferred to a Hybond ECL (enhanced chemiluminescence) polyvinylidene membrane (Amersham Biosciences, Piscataway, NJ, USA). The membranes were blocked in a 5% nonfat dry milk solution in phosphate-buffered saline containing 0.1% Tween 20. The protein was probed with phospho-Ezrin, Radoxin and Meosin (pERM), total ERM, RhoA, -catenin, phosphor-PTEN (phosphatase and tensin homologue), glyceraldehyde 3-phosphate dehydrogenase (GAPDH), and phospho-PDK1 (1:1000 rabbit polyclonal antibody, Cell Signaling Technology, Danvers, MA, USA). After incubation in enhanced chemiluminescent solution, the bands were detected by a Chemi-Doc image system (Bio-Rad Life Science, Hercules, CA, USA) [12].

### 2.5. Real-Time Polymerase Chain Reaction

RNA was purified from 50 mg of heart tissue from the non-infarcted zone using Tri-sol RNA extraction reagents (BMS, Amherst, NY, USA). For RNA quantification, the complementary DNA (cDNA) was synthesized using 1 μg of RNA through a reverse transcription reaction using an oligo-dT primer reverse-transcription kit (Intron Biotech Co., SeongNam, Korea). A real-time polymerase chain reaction (PCR) was performed in an ABI Prism 7000 Sequence Detection System using the SYBR Green® Fluorescence Quantification System (Applied Biosystems, Warrington, UK) to quantify amplicons. The standard PCR conditions were 95 °C for 10 min, then 40 cycles at 95 °C for 30 s, and 60 °C for 30 s, followed by a standard denaturation curve. The sequences of the transforming growth factor-β (TGF-β); forward 5’-TGA TAC GCC TGA GTG GCT GTC T-3’, reverse 5’-CAC AAG AGC AGT GAG CGC TGA A-3’, and GAPDH; forward 5’- ATC AAC GAC CCC TTC ATT GAC C-3’ and reverse 5’-CCA GTA GAC TCC ACG ACA TAC GC-3’ primers were designed using the Primer Express^®^ software (Thermo Fisher Scientific, Frankfurter, Germany) with nucleotide sequences found in the GenBank database.

### 2.6. Microarray Analysis

RNA isolated from the apical heart excluding scar tissue was labeled with either cyanine 3-CTP (Cy3) or cyanine 5-CTP (cy5) (PerkinElmer, Boston, MA, USA) using a Low RNA Input Fluorescent Linear Amplification Kit (Agilent Technologies, Santa Clara, CA, USA) and hybridized to an Agilent rat whole-genome array. RNA samples from 3 rats in each group were pooled and hybridized to 1 chip. Three chips per group were used for the MI-only and MI + Fima groups. The arrays were scanned at 2 different intensities, and the images were analyzed for background corrections. Both the MI-only and MI + Fima samples were co-hybridized with RNA from the starting point, and a dye swap was performed. The arrays were normalized, and the differential gene expression was analyzed using the R and Bioconductor-based method LIMMA [13,14,15]. After the genes were sorted by expression level, enrichment scores were assigned to 572 different gene sets curated in the DAVID Bioinformatics Resources 6.8 database (https://david.ncifcrf.gov). Gene sets with high absolute values of the enrichment score are molecular pathways or gene ontologies consisting of upregulated or downregulated genes and are differentially regulated pathways. *P*-values of the enrichment scores were calculated after permuting the class labels of each experimental condition, and gene sets with *P*-values < 0.05 were extracted [15].

### 2.7. Statistical Analysis

The data are reported as means and standard deviations. The one-way analysis of variance or the Kruskal–Wallis test was used to compare the mean values of the different groups whenever appropriate. Values of *P* < 0.05 were considered statistically significant. The statistical analysis was performed with SPSS Statistics (Version 19.0, IBM SPSS Inc., Chicago, IL, USA).

## 3. Results

### 3.1. Animals

Overall, 7, 13, and 16 rats were included in the sham group, MI-only group, and MI + Fima group, respectively (Figure 1).

### 3.2. Echocardiographic and Hemodynamic Findings

**Figure 2:**
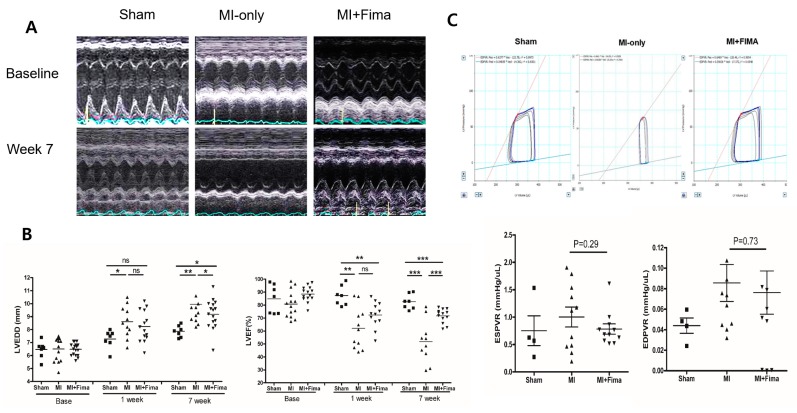
The anatomy and hemodynamics: (**A**) The chamber size measurement in M-mode, (**B**) the chamber size and ejection fraction of the rats during the experiment, and (**C**) the pressure–volume loops 7 weeks after MI. ■, sham; ▲, MI; ▼, MI + Fima. The MI + Fima group showed a smaller left ventricular volume and larger end-systolic pressure–volume relation (end-systolic pressure–volume relation, red line) compared with the MI-only group. EDPVR, end-diastolic pressure–volume relation; EDP, endo-diastolic pressure; ESPVR, end-systolic pressure–volume relationship; Fima, fimasartan; MI, myocardial infarction; LVEDD, left ventricular end-diastolic diameter; LVEF, left ventricular ejection fraction. The *P*-values represent * *P* < 0.05, ** *P* < 0.01, and *** *P* < 0.001. The results are expressed as mean ± SEM (Standard Error Mean).

Echocardiographic findings are summarized in Table 1. At baseline, there was no difference in echocardiographic measurements among the three groups. At 7 weeks, the MI-only group had a larger mean LVEDD and smaller mean EF than the sham group. Interestingly, the MI + Fima group had a smaller mean LVEDD (9.14 ± 1.11 mm vs. 9.91 ± 1.43 mm, *P* = 0.045), a smaller IVSd (0.90 ± 0.35 mm vs. 1.14 ± 0.38 mm, *P* = 0.072), but greater EF (66.3 ± 12.5% vs. 51. 3 ± 14.8, *P* = 0.002) than the MI-only group (Figure 2A,B).

The results of a hemodynamic assessment conducted at week 7 are summarized in Table 2. Overall, the MI-only and MI + Fima groups had larger mean end-systolic volume values and smaller mean SV values than the sham group. The MI + Fima group had a greater mean stroke volume (76.1 ± 28.5 μL vs. 53.2 ± 19.6 μL, *P* = 0.04) and a greater mean maximal pressure − dP/dt than the MI group with a marginal significance (4839.2 ± 1776.5 mmHg/s vs. 3614.0 ± 1004.9 mmHg/s, *P* = 0.06). Although the MI + Fima group had a numerically lower end-systolic pressure–volume relation and end-diastolic pressure–volume relation than MI-only group, the difference was not statistically significant (Figure 2C).

### 3.3. Heart weight and Infarct Size

**Figure 3:**
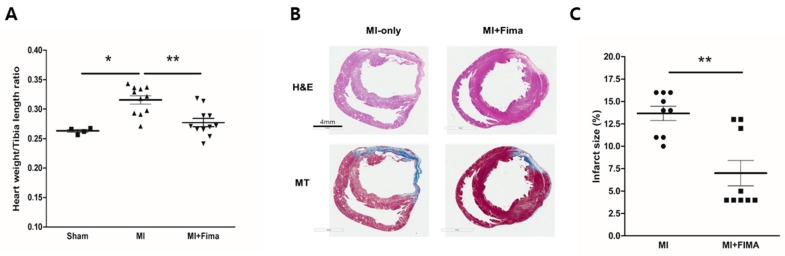
A histologic examination of the explanted hearts: (**A**) Cardiac hypertrophy was assessed as the heart weight/tibia length ratio. Fimasartan attenuated the induction of cardiac hypertrophy after MI. ■, sham; ▲, MI; ▼, MI + Fima. (**B**) The hematoxylin and eosin (H&E) and Masson trichrome (MT) staining showed a smaller infarct size and fibrosis in the MI + Fima group. (**C**) The MI + Fima group (■) had a smaller infarct size than the MI-only group (•). The *P*-values represent * *P* < 0.05, and ** *P* < 0.01. The results are expressed as mean ± SEM.

After the organ harvest, the heart weights and tibia lengths were measured. The mean heart weights were 529 ± 47.9 mg, 517.6 ± 32.9 mg, and 495.3 ± 45.3 mg in the sham, MI, and MI + Fima groups, respectively. After an adjustment for tibia length, the MI-only group had the highest heart weight/tibia length ratio, whereas there was no difference between the sham and MI + Fima groups, suggesting that fimasartan prevents the progression of LV remodeling after acute MI (Figure 3A).

Cross-sectional samples from the MI-only and MI + Fima groups showed myocardial infarction. However, the MI + Fima group had a smaller infarct size than the MI-only group (13.67 ± 0.7993% vs. 7 ± 1.424%; *P* < 0.01) (Figure 3B,C).

### 3.4. Transcriptional Changes in Molecular Pathways

**Figure 4:**
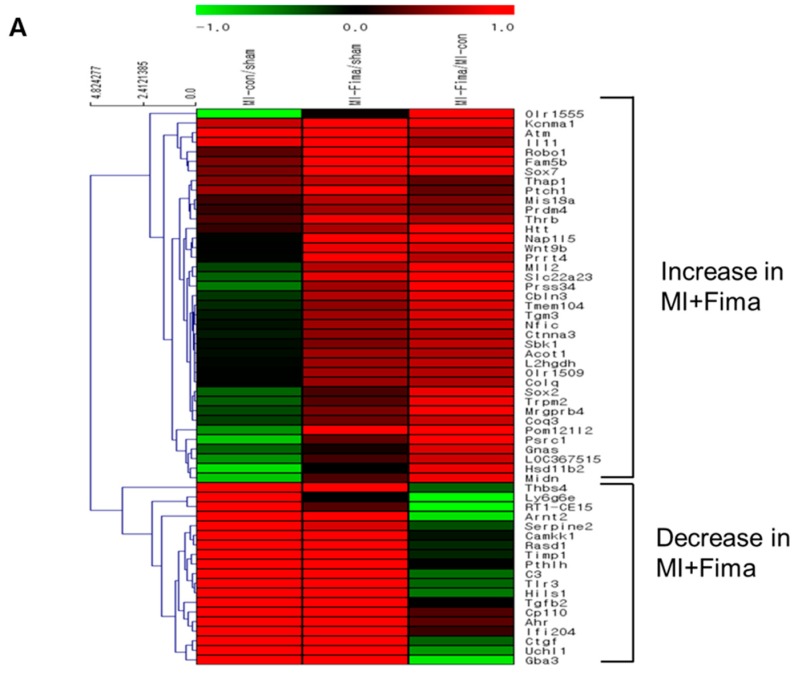

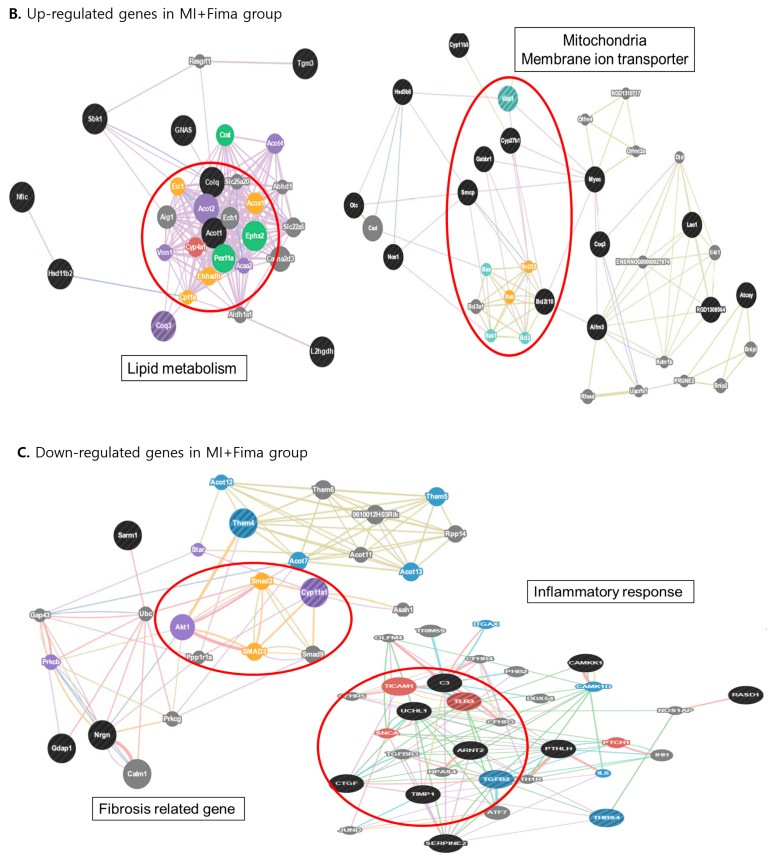
The transcriptional changes in the molecular pathways: (**A**) The microarray analysis in MI-only and MI + Fima group. In the MI + Fima group, 39 genes were upregulated and 19 genes were downregulated. (**B**) The clustering analysis showed the upregulation of lipid metabolism and mitochondria membrane ion transporter gene sets in the MI + Fima group. (**C**) The inflammation and fibrosis inducing genes were downregulated in the MI + Fima group.

A total of 58 of 44,000 genes showed different expression levels (≥2-fold) between the MI + Fima and MI-only groups. In the MI + Fima group, 39 genes were upregulated and 19 were downregulated (Figure 4A). For biological interpretation, we performed the gene set enrichment analysis using a web-based system (DAVID) to investigate the transcriptional changes in the molecular pathways or gene ontologies. Figure 4B,C shows a network representation of the gene sets that were enriched in different gene regulations in the MI + Fima group and that may be associated with the pharmacological effect of fimasartan in MI. Lipid metabolism and mitochondrial membrane transporter genes, which are essential for improving cardiac myocyte survival, such as acyl-CoA thioesterase 1 (Acot1), acylCoA oxidase 1 palmitoyl (Acox1), and ubiquinol cytochrome c reductase (Uqcrfs1), were upregulated, whereas fibrosis and inflammatory response genes, which induce cardiac remodeling in cardiac damage, such as SMAD family member 2, 3, and 9 (SMAD2, 3, 9), TIMP metallopeptidase inhibitor 1 (TIMP1), connective tissue growth factor (CTGF), toll-like receptor 3 (TLR3), and TGF-β2, were downregulated in the MI + Fima group.

### 3.5. Effect of Fimasartan on Molecular Biology Level

**Figure 5:**
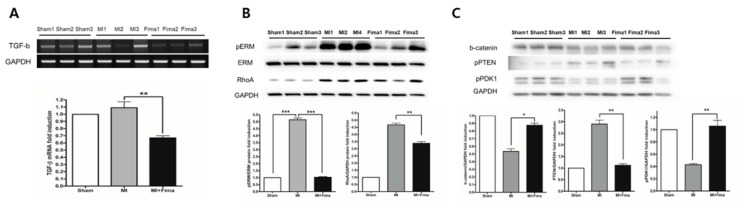
The change in the cardiomyocyte remodeling and survival signaling with fimasartan: (**A**) TGF-beta was significantly decreased in the MI + Fima group. (**B**) The cardiac remodeling regulating molecule ERM and RhoA kinase activity were downregulated in the MI + Fima group. (**C**) The cell survival regulating molecule -catenin, PTEN, and PDK1 activity were upregulated in the MI + Fima group. The *P-*values represent * *P* < 0.05, ** *P* < 0.01, and *** *P* < 0.001. The results are expressed as mean ± SEM. ERM, Ezrin, Radoxin and Meosin; PTEN, phosphatase and tensin homologue; GAPDH, glyceraldehyde 3-phosphate dehydrogenase; PDK1, Pyruvate Dehydrogenase Kinase 1.

TGF-β is known to be involved in fibrosis and LV remodeling; therefore, here, we examined its expression. Consistent with the findings in the microarray analysis, the TGF-β expression was lower in the MI + Fima group than in the MI-only group (Figure 5A). Cardiac remodeling regulating signal molecule Ezrin, Radixin, and Moesin (ERM) and RhoA kinase activity were significantly lower in the MI + Fima group than in the MI-only group (Figure 5B). The MI + Fima group had a strongly upregulated cardiac myocyte survival signaling, such as β-catenin, PTEN (decrease phosphorylation), and PDK1 (increase phosphorylation) activity (Figure 5C).

## 4. Discussion

The purpose of this study was to evaluate the effect of fimasartan treatment on cardiac remodeling, function, and hemodynamics after acute MI. We showed that fimasartan treatment mitigated cardiac remodeling and dysfunction by suppressing inflammation and fibrosis-related gene expression in rats after MI. Fimasartan is a new angiotensin II receptor antagonist with a high selectivity for the AT_1_ receptor subtype and a lower dissociation constant, implying a long and stable effect. Fimasartan is a potent blood pressure lowering drug in patients with hypertension [16,17,18]. However, its effect on cardiac remodeling after MI has not been well-established.

In this study, the fimasartan treatment reduced the positive remodeling in rats after MI. Rats treated with fimasartan had a smaller heart size, suggestive of mitigated cardiac remodeling. In the histological sections, fimasartan significantly reduced fibrosis in the infarct zone. Furthermore, rats treated with fimasartan had a greater stroke volume and maximal dp/dt, indicating a better preserved systolic function. Our study findings are in line with those of previous studies and current practice guidelines recommending the renin-angiotensin-system inhibitor treatment in patients after MI [19]. To explain the effect of fimasartan on cardiac remodeling and hemodynamics, we performed microarray and western blot assays and examined the genetic expression and transcription of signaling pathways known to be involved in cardiac remodeling and cell survival. 

In the microarray analysis, we showed that the expression of genes involved in lipid metabolism and mitochondrial membrane ion transportation were upregulated, and those involved in fibrosis and inflammation were downregulated with fimasartan treatment. Such changes in gene regulation may be responsible for the attenuated cardiac remodeling and improved cardiomyocyte survival noted here. In the western blot assay, the fimasartan treatment suppressed phospho-ERM and RhoA, which are involved in fibrosis and LV remodeling in the remote myocardium after ischemia. Fimasartan also reduced the activity of Rho kinase, which is involved in the pathogenesis of vascular remodeling [20,21,22,23,24], cardiac hypertrophy [25,26], and the release of proinflammatory cytokines [26]. The reduced expression of TGF-β in the MI + Fima group may be explained by the inhibition of RhoA kinase. Indeed, TGF-β is associated with fibrosis formation and cardiomyocyte hypertrophy after MI.

Wnt/β-catenin is the main signaling pathway in the induction of compensatory hypertrophy [27] and the inhibition of the apoptosis of cardiomyocytes [28,29]. The activity of β-catenin was downregulated by GSK3; therefore, many GSK3 inhibitors are currently under investigation as potential therapeutic agents to maintain cardiomyocyte integrity after MI. In the present study, the fimasartan treatment reduced PTEN phosphorylation and increased the PDK1 phosphorylation, which could enhance Wnt/β-catenin activity. This may explain the effect of fimasartan on LV remodeling and heart function. Nonetheless, whether the study results can be directly applied to humans needs further investigation.

## 5. Conclusions

Fimasartan mitigates cardiac remodeling and systolic dysfunction in rats after MI. However, it is unknown whether the results of this animal experiment can be extrapolated to humans. Therefore, clinical trials are necessary to evaluate the effect of fimasartan in humans after MI.

## Figures and Tables

**Table 1:** The echocardiographic findings.

Variables	Sham Group (*n* = 7)	MI-only Group (*n* = 13)	MI + Fima Group (*n* = 16)	*P*-Value
Baseline				
LVEDD, mm	6.46 ± 0.66	6.50 ± 0.91	6.48 ± 0.47	0.99
LVESD, mm	3.16 ± 1.21	3.51 ± 1.14	3.04 ± 0.65	0.39
IVSd, mm	1.27 ± 0.15	1.14 ± 0.10	1.22 ± 0.11	0.034
LVPWd, mm	1.36 ± 0.13	1.19 ± 0.11	1.31 ± 0.13	0.007
EF, %	84.7 ± 11.1	80.8 ± 10.2	87.3 ± 6.31	0.127
Week 1				
LVEDD, mm	7.27 ± 0.70	8.22 ± 1.31	8.29 ± 1.09	0.122
LVESD, mm	3.39 ± 1.18	5.53 ± 1.82	5.42 ± 1.15	0.006
IVSd, mm	1.34 ± 0.11	1.01 ± 0.21	1.05 ± 0.29	0.014
LVPWd, mm	1.36 ± 0.13	1.35 ± 0.12	1.31 ± 0.16	0.674
EF, %	87.3 ± 7.61	66.4 ± 16.4	69.1 ± 9.99	0.003
Week 7				
LVEDD, mm	7.87 ± 0.44	9.91 ± 1.43	9.14 ± 1.11	0.002
LVESD, mm	4.19 ± 0.68	7.61 ± 1.84	6.14 ± 1.38	<0.0001
IVSd, mm	1.39 ± 0.16	0.90 ± 0.35	1.14 ± 0.38	0.002
LVPWd, mm	1.47 ± 0.17	1.41 ± 0.21	1.36 ± 0.18	0.411
EF, %	82.6 ± 5.74	51.3 ± 14.8	66.3 ± 12.5	<0.0001

LVEDD, left ventricular end-diastolic diameter; LVESD, left ventricular end-systolic diameter; IVSd, interventricular septal thickness at diastole; LVPWd, left ventricular posterior wall thickness at diastole; EF, ejection fraction.

**Table 2:** The hemodynamics measurements at week 7 after a myocardial infarction.

Variables	Sham Group (*n* = 4)	MI-only Group (*n* = 11)	MI + Fima Group (*n* = 11)	*P*-Value
HR, bpm	264.3 ± 79.1	306.4 ± 55.8	307.9 ± 47.3	0.386
ESV, μL	241.9 ± 70.8	337.2 ± 67.0	344.7 ± 50.8	0.023
EDV, μL	343.6 ± 114.5	368.2 ± 64.6	377.8 ± 55.6	0.707
SV, μL	120.8 ± 59.9	53.2 ± 19.6	76.1 ± 28.5	0.005
+dP/dt, mmHg/s	5456.0 ± 402.7	3957.3 ± 907.7	4800.8 ± 1354.5	0.054
−dP/dt, mmHg/s	4990.3 ± 1030.7	3614.0 ± 1004.9	4839.2 ± 1776.5	0.094
ESPVR, mmHg/μL	0.75 ± 0.54	1.00 ± 0.61	0.78 ± 0.31	0.509
EDPVR, mmHg/μL	0.04 ± 0.01	0.09 ± 0.06	0.08 ± 0.07	0.512

Data are mean ± SD. The *P*-value was calculated from an one-way analysis of variance test among the three groups, and then, an unpaired student’s *t*-test was performed between two groups. MI indicates myocardial infarction; HR, heart rate; ESV, end-systolic volume; EDV, end-diastolic volume; SV, stroke volume; ESPVR, end-systolic pressure–volume relationship; EDPVR, end-diastolic pressure–volume relationship.
